# Visualising Conversation Structure across Time: Insights into Effective Doctor-Patient Consultations

**DOI:** 10.1371/journal.pone.0038014

**Published:** 2012-06-05

**Authors:** Daniel Angus, Bernadette Watson, Andrew Smith, Cindy Gallois, Janet Wiles

**Affiliations:** The University of Queensland, St. Lucia, Queensland, Australia; The University of Hong Kong, Hong Kong

## Abstract

Effective communication between healthcare professionals and patients is critical to patients’ health outcomes. The doctor/patient dialogue has been extensively researched from different perspectives, with findings emphasising a range of behaviours that lead to effective communication. Much research involves self-reports, however, so that behavioural engagement cannot be disentangled from patients’ ratings of effectiveness. In this study we used a highly efficient and time economic automated computer visualisation measurement technique called Discursis to analyse conversational behaviour in consultations. Discursis automatically builds an internal language model from a transcript, mines the transcript for its conceptual content, and generates an interactive visual account of the discourse. The resultant visual account of the whole consultation can be analysed for patterns of engagement between interactants. The findings from this study show that Discursis is effective at highlighting a range of consultation techniques, including communication accommodation, engagement and repetition.

## Introduction

Effective communication between health professionals and their clients is a critical part of healthcare [1], [2], [3]. Poor communication in the health context can result in adverse outcomes for patients [4]. In addition, effective communication is associated with patient adherence to treatment regimes and with improved health outcomes [5].

Over the past decade there has been a shift from a focus on health professionals’ role in the consultation to that of patients’ communication [6]. Watson and Gallois have argued that understanding both perspectives is important, because communication is a dynamic two-way interaction [7]. Their findings indicate that certain patterns of communication behaviour by health professionals, such as joint engagement in the conversation and emotional support, are good predictors of patient satisfaction [8]. The current paper combines this theoretical approach with a software tool that simplifies the analysis of actual interactions.

A key difficulty in examining health communication interactions is the in-depth analysis required to get a detailed portrayal of verbal positioning by speakers. Heritage and Maynard [9] outline two key approaches to the analysis of conversation, which help to clarify the role of software like Discursis: process analysis and microanalysis [9]. Process analysis involves developing a coding scheme to characterise each speaker’s performance. Roter’s Interaction Analysis System (RIAS) [10], and Bales’ Interaction Process Analysis [11], [12] exemplify this technique. They produce systematic coding of all utterances by applying a generic and comprehensive categorisation system. They focus on turn-by-turn conversational behaviour (e.g., questions, interruptions, words of reassurance), and some systems (but notably not Conversation Analysis [13]) tally up the amount of each type of communication behaviour. Process analysis requires many hours of specialist training and experience to enable accurate coding of behaviour. Heritage and Maynard [9] note that this approach focuses on the coding rather than the content, which is a weakness of the technique.

Microanalysis (e.g., conversation analysis, see [13]) addresses this limitation by examining every detail of the interaction with respect to the context and cultural meaning of the specific encounter. However, this approach does not lend itself to linking conversational styles with outcomes. In health care, the connection between communication behaviours and outcomes such as satisfaction and treatment adherence are central to the research endeavour. If there is interest in, for example, whether a patient’s initial concerns are addressed in a systematic way through the consultation, microanalysis must rely entirely on the interpretive skills of the researcher. Furthermore, neither process analysis nor microanalysis provides visualisations to highlight the ways in which interactants share information and engage with each other, an area cited as being in need of improvement [14]. For further exposition of the different approaches to analysing medical interactions, see [9].

Visual Text Analytics is a growing sub-field of Information Visualisation concerned with generating visual accounts of text data [15]. These computational techniques are not aimed at replacing traditional analysis and methodologies; instead, they are aimed at augmenting existing approaches through the provision of additional insights into the data that are difficult to obtain through non-visual means. In this paper we explore the application of a visual text analytic approach, Discursis [16], [17], to a series of health communication interactions, and we interpret the visualisations in the context of Communication Accommodation Theory (CAT) [18]. Discursis was designed specifically for assisting conversation transcript analysis, and this paper details the first application of Discursis to medical transcripts.

Discursis is a visualisation system that shows the temporal structure of a conversation, by representing the time series comprising each speaker’s turn as well as the concepts shared between turns. Discursis automatically builds an internal language model from an input text, tags each temporal unit (a single turn in the context of a conversation) based on the conceptual content, and generates an interactive visual representation of the input text. The Discursis visual representation enables an analyst to overview an entire text quickly to examine the turn taking dynamics (who speaks when and for how long), the thematic content of the text over time, and regions of thematic coherence over short (turn-by-turn), medium (typically 10 temporal units) and long (whole conversation) time scales. Discursis is useful for locating periods of conversation where participants engage in similar topics or repeat their own content, in addition to periods that lack topical coherence. A more detailed description of the use of Discursis in this paper is given in Section 2.4, and the overall system is described in File S1, for details of the underlying algorithms, see [16], [17].

### Study Aims

In this study we used the Discursis information visualisation technique to investigate how short (turn-by-turn), medium (10 turns) and long-term (whole consultation) engagement patterns between doctors and patients are related to task and rapport building behaviour. We aimed to identify visual features present in Discursis visualisations and attribute these features to task and rapport building in a doctor/patient consultation context, and determine the suitability of the Discursis technique to the analysis of medical consultation transcripts.

### Background Literature

In this paper we combined robust communication theory used in the healthcare context and a new visualisation technique (Discursis) to capture the dynamics of the ongoing interaction. Discursis has the facility to be both a practical and theoretical tool. It can identify how well each interactant is included in the consultation and the opportunities each has to engage in topic sharing and topic elaboration. These facets of communication are important for all health practitioners as they try to tease out their clients’ health concerns. For researchers, Discursis contributes to applying and extending communication theory to actual conversational behaviour. In this paper, we discuss both training and theoretical development.

#### Clear outcomes from simulated interaction

Discursis has been used previously in the analysis of conversation transcripts from television interviews, where genre-specific patterns of interaction were identified and linked to interactants’ behaviour [16], [17]. These findings led us to the use of Discursis in the health context.

#### Effective doctor/patient consultation: communication accommodation

An efficient and effective doctor/patient consultation balances two objectives, task focus and rapport building [19]. Task focus relates to discussion of medically relevant details, whereas rapport building relates to the socio-emotional relationship that develops between a physician and a patient. Task focus and rapport building should not be considered as mutually exclusive processes, although in many practical situations an increase in efficacy in one will lead to a decrease in the other [20]. A rapport-rich but task-deficient consultation may appear to contain good engagement between the doctor and patient and can leave a patient feeling satisfied with the outcome. Nevertheless, these consultations often lack a concrete diagnosis and clear treatment outcome [20]. Conversely, in a rapport-deficient consultation the doctor may fail to engage with the patient and thus struggle to obtain the details necessary for obtaining a diagnosis, or may leave a patient unengaged, making treatment less effective [21].

According to Platt and Gordon [22] the keys to an efficient and effective patient interview are engagement and enlistment. Engagement concerns how much the doctor and patient share the health narrative, and enlistment is defined as how well a patient follows recommendations. In their 2004 field guide to patient interview techniques, Platt, Gordon and their colleagues identify several key steps to guide the physician [22]. Their guidelines align with researchers using communication accommodation theory [18], who describe five accommodative communication strategies required for an effective health consultation [7], [23]. The strategies include *approximation*, which (when it is appropriately accommodative) involves matching of one interactant’s behaviour by another, on verbal (language, same-saying, style) or non-verbal (vocal features and qualities, gesture, etc.) channels. In addition, the *interpretability* strategy involves clear language by the doctor to ensure that the patient engages and understands the consultation process. The third strategy, *discourse management*, involves the doctor ensuring that the patient has an opportunity to engage in the interaction through expressing his or her concerns and viewpoint, and by sharing in formulation of the topics. *Emotional expression* occurs when the doctor recognises how much reassurance the patient requires, and along with discourse management assists in building rapport. Finally, accommodative *interpersonal control* occurs when the patient is not constrained in the role of patient by a doctor who dominates the consultation, but rather is treated as an individual experiencing health concerns that affect his or her life.

## Methods

### Information Visualisation: Visual Text Analytics

Information visualisation techniques can produce interesting and informative graphics from a variety of input media. The choice of visualisation technique generally depends on the temporal and spatial characteristics of the input data, the perceptual and cognitive capabilities of the users, and the analytic goals. Visual text analytic techniques are a class of information visualisation that generate visual accounts of text data. As one example, Leximancer™ [24], [25] is a commercially-available visual text analytic system that represents the prominent concepts from an input text corpus on a two-dimensional map, with theme circles grouping coherent sections of the map into clusters, and a spanning tree connecting related concepts. Leximancer focuses on spatial aspects of input text (how concepts extracted from the text are related to each other) and has been used previously with success for analysing health communication [26], [27].

**Figure 1:**
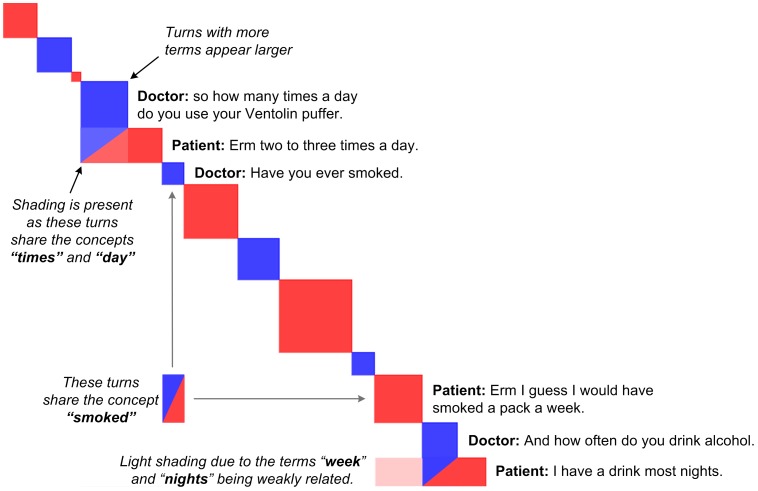
Conceptual Recurrence Plot of 13 utterances and 4 corresponding recurrence elements from a Doctor/Patient consultation. The Patient is coloured red and the Doctor is coloured blue. Conceptual recurrence between the Patient and the Doctor is indicated by a half/half coloured square, and self-recurrence is in the speaker’s own colour.

In contrast to the spatial (or semantic) focus of many text analytic tools, the Discursis [16] technique was specifically designed for analysis of the temporal (or episodic) aspects of communication, and extends an existing visualisation technique called *recurrence plotting*, which is used to display and identify trends within time series data [28]. Discursis displays a conversation diagonally turn-by-turn, and analyses the extent to which people are using similar topics or concepts, repeating their own topics or concepts, or are unrelated. If any two turns in a conversation contain similar concepts, then the corresponding off-diagonal element is shaded to indicate the degree of conceptual similarity. A brief description of the Discursis technique is included in File S1, and an example Discursis plot is shown in Figure 1.

### Data

Two data sets of physician/patient consultations were analysed in this study: short scenarios adapted for training doctors (training dataset) and complete clinical sessions between a doctor and patient (clinical dataset). The three examples in the training dataset were selected because they contain extremes in behaviour (see below). The three examples from the clinical dataset were transcripts from actual doctor and patient consultations.

#### Training data

The first dataset contained 12 consultations, provided by Marcus Watson from the Queensland Health Skills Development Centre. These training scenarios are used in communication skills training exercises to highlight effective and ineffective communication in a clinical setting, and were inspired by real-life doctor/patient interactions. The consultation variations were developed from an actual consultation that had been video-recorded. The original consultation was edited in one variation to include communication that showed the doctor not being task focused; for example, discussing mutual interests rather than the patient’s symptoms and associated information. In another variation, the doctor was too directive and did not provide the patient with an opportunity to elaborate. These changes were specifically designed to incorporate recognised issues in medical interactions. The three consultations demonstrate exemplars of good and poor task focus, and good and poor rapport building, and serve as contrasting examples for later analysis of real-life clinical datasets.

The situation modelled in the training consultations is a patient (David) presenting with symptoms including dizziness and shortness of breath. As things turn out, the cause of David’s malaise is a compound found in paint that he is using at his home and workplace. Neither David nor the doctor has this information prior to the consultation. Elicitation of this information should lead to a correct diagnosis, whereas failure to uncover this information will most likely result in a failure to identify the root cause of David’s malaise. In the modelled situation the physician and patient have never met, as David has recently moved to a new city. Thus, the doctor must uncover multiple seemingly unconnected details about the patient’s work and home life to determine the root cause of the illness.

Three consultations from this dataset were analysed in detail for this study, labelled Training #1, Training #2 and Training #3. These consultations were selected as they contain three combinations of good/poor task focus and rapport.

#### Training #1

Good task focus and good rapport. In this consultation the doctor is able to arrive at the correct diagnosis and enlists the patient in the steps required to treat the problem.

#### Training #2

Poor task focus and poor rapport. In this consultation the doctor is unable to arrive at a correct diagnosis, does not engage the patient’s concerns and does not ask the right medically relevant questions.

#### Training #3

Poor task focus but good rapport. In this consultation the doctor engages the patient well and builds good rapport, but this rapport building comes at the expense of locating important medical information, and therefore the doctor does not achieve a correct diagnosis.

#### Clinical data

The second dataset contained consultations that were recorded as part of larger study investigating effective communication between health professionals and patients. Although a range of health professionals from the disciplines of nursing, physiotherapy, occupational therapy and speech pathology participated in the larger study, for this study we selected three examples of doctor-patient consultations to provide a comparison with the three training consultations. Owing to the fact that all patients reported high satisfaction with their consultations, choosing transcripts that received patient ratings of a bad interaction was not possible. Questions rated by patients about the interactions were developed from CAT and reflected aspects of the medical encounter, so we looked for patients who rated their consultation as less positive than the majority in order to find subtle variability in the dataset (see [7]). We found three examples where patients had provided less positive ratings on one or more of the following items: he or she had felt constrained by time factors during the consultation, felt that seeing another doctor at the next consultation might be useful, or felt that quality of life had not improved since seeing the doctor. Patients consented to be part of a study on doctor-patient communication, were provided with information about the study, and signed a consent form agreeing to the consultation being recorded. Before the consultation the doctor and patient independently completed a brief questionnaire. Patients answered questions about their health, their beliefs about participation in health, and their expectations in a medical consultation. Doctors also completed a questionnaire about their knowledge of the patients and their communication expectations in a consultation. At the commencement of the consultation a researcher started the recording equipment and left the room. At the conclusion of the consultation each patient and doctor independently completed a questionnaire about perceptions of the consultation. One month after the consultation, each patient received a follow-up questionnaire, which had the same items as the post consultation questionnaire and also asked patients how much they had adhered to treatment. Three consultations from this dataset were analysed in this study, labelled Clinical #1, Clinical #2 and Clinical #3.

#### Clinical #1

HP18 was a female doctor in general practice who examined a 51 year old woman (Pat30) with multiple health problems. On this occasion the patient had presented for a pap smear.

#### Clinical #2

HP22 was a male doctor in general practice who examined a 46 year old woman (Pat38) who was a foster carer. The patient presented with an ear problem.

#### Clinical #3

HP22 also examined a 39 year old woman (Pat37) who was a pensioner. The patient presented with liver problems, as well as other serious health issues.

## Results

### Training Consultations

Using Discursis, we analysed each of the three training consultations separately; each consultation involved a conversation between the doctor and David (patient). First, we looked at the extent of intra-speaker and inter-speaker concept similarity (in CAT terms, accommodative approximation via same-saying on concepts) on a turn-by-turn basis. This allowed an assessment of short-term conceptual engagement between the doctor and patient, as well as the extent of conceptual consistency for each speaker. Next, we examined the conversations in 10-turn blocks, which allowed us to assess engagement and similarity across blocks of speech. Finally, we examined each conversation as a whole. We expected the consultation with good task and good rapport (Training #1) to contain stronger engagement at each level than the consultation with poor task and poor rapport (Training #2). We also expected Training #1 to contain more engagement than the consultation with poor task and good rapport (Training #3) in terms of medical content and attention to David’s medical problems, although we expected good engagement around non-medical topics in Training #3.

**Figure 2:**
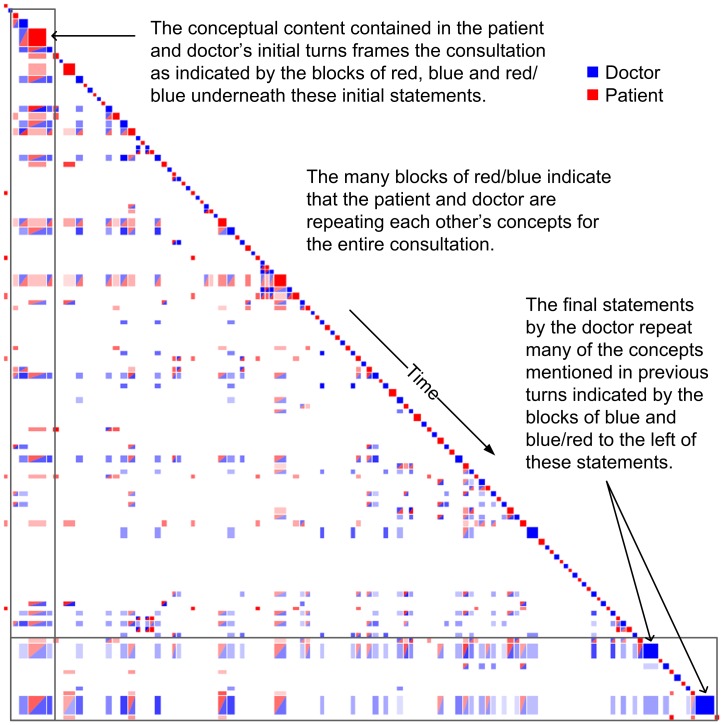
Features of a good doctor/patient consultation. Strong engagement between the doctor (blue) and patient (red) is observed throughout the whole consultation, observable by the two-colour recurrence blocks.

For Training consultation #1, turn-by-turn analysis showed the patient engaging with statements by the doctor and the doctor engaging with statements by the patient; that is, there was significant approximation or repetition of concepts across speakers. Such patterns of engagement can be seen as two-colour squares connected to the diagonal, which only occur when the doctor and patient repeat concepts mentioned in the turn immediately prior to their current turn (see Figure 2). At the 10-turn level, this impression of strong engagement around the patient’s medical problems was reinforced. For example, at the half way point of the consultation the doctor engaged strongly with the patient around the concept of drinking. This engagement was found by looking for sections of connected recurrence, which manifest as many red, red/blue, and blue blocks next to each other that are close to the diagonal. At the level of the whole conversation, the level of engagement was also high, particularly between the opening turns by the patient and the remainder of the consultation, and the final turns by the doctor and the turns that had appeared earlier. This means that both doctor and patient accommodated to the patient’s initial presentation of the problem, and this stance of approximation continued throughout the conversation. Thus, several stripes of vertical recurrence can be seen stemming from the patient’s initial turns (highlighted in Figure 2). These vertical stripes indicate that the conceptual content of these early turns was repeated throughout the remainder of the consultation. The conceptual content of these early turns recurred with both the patient’s own statements (red squares) and the doctor’s statements (red and blue squares) throughout the remainder of the consultation. This feature also indicates that these early turns framed much of the later discussion. The opening exchange of Training #1 is reproduced below, with the text of the large red square at the head of one of the vertical stripes in Figure 2 indicated in bold below:

Doctor: Good morning David, I am Dr Vivien Ling. How are you today.

David: Alright, I guess.

Doctor: This is your first visit to our clinic.

David: Yes, my family moved here from Hobart when er Karin got a job with Powerlink last year.

Doctor: Now David you’ve been having some problems ah with vertical dizziness. Errmm you’ve written me a letter and so’s your doctor in Hobart about your problem. Would you like to tell me about er the particular trouble you’ve been having.

**David: Yes, well I’ve had dizzy spells as such for oh many years erm in fact looking back I’d say probably from when my children were very young which would be more than four years ago. Dizziness in that er in motion particularly. I’ve always been motion sickness, sea sickness, air sickness erm. It’s been getting progressively worse in the last few years. I’ve been treated for vague ear infections and so on which may have caused the dizziness but in the last twelve months and particularly in the last six months it’s been getting so bad that I’m almost living with dizziness all the time.**

The stripes of blue and blue/red coloured horizontal recurrence stemming from the doctor’s closing statements suggest that these statements summarise many of the key concepts raised throughout the entire consultation. Such a feature may not be present if a doctor does not offer a conclusive or substantive diagnosis. One of these statements is reproduced below:

Doctor: David correct me if I am wrong. Er you’ve been suffering asthma since you started working as a panel beater. Over the last few years you have done more spray painting work without protective equipment. Since you have moved to Brisbane you have been doing a lot more spray painting in your garage. Your dizziness has increased since you started doing more of this work at home. Er David you have no family history of dizziness and you have suffered no head injuries.

**Figure 3:**
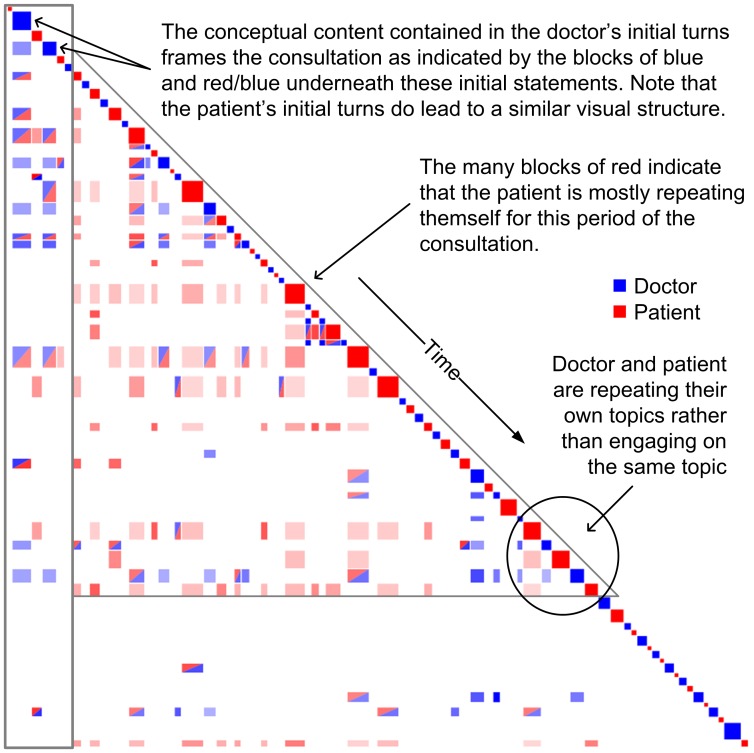
Features of a poor doctor/patient consultation. Good engagement between the doctor (blue) and patient (red) is witnessed early in the consultation; however there after the consultation degrades over time as the patient (red) begins to repeat themself.

For training consultation #2, turn-by-turn analysis showed a limited amount of engagement, particularly in the latter half of the consultation where not a single red/blue block is observed next to the diagonal (see Figure 3). At the 10-turn level, this impression of limited engagement around the patient’s problems is reinforced, one example being near the end of the consultation where the doctor and patient are seen to repeat their own concepts but not engage with each other’s concepts (that is, they do not approximate but maintain their own concepts). Such behaviour presents as a checkerboard style pattern due to participants repeating their own concepts but not the other participant’s concepts. In this example the doctor is curious about the patient’s home life, but the patient is concerned that his dizziness may be caused by a tumour. The doctor in this instance failed to engage the patient about his concerns over a tumour and thus was unable to get a straight answer to repeated questioning:

Doctor: How is your home life.

David: Erm good I suppose. Erm Karin and me fight sometimes but in general it’s good. I don’t get much sleep but. Cause of the kids and the dizzy spell. You don’t think is a tumour do you.

Doctor: Was your home life the same when you lived in Hobart. Did you get more sleep.

David: Yea, I suppose I did Clair didn’t like the move. Er we had to pull her out of her school and er she misses her friends. Doc my Dizziness is getting worse I am not going to die am I.

Doctor: Can you please focus on answering my questions. Is there anything at home or at work that you think might be causing the dizziness.

David: Erm nothing I can think of er na nothing. That’s why Karin thinks it’s a tumour. They can fix them these days can’t they doc.

In the block presented here, accommodative communication is not evident. First, there is little or no approximation at the conceptual level. In addition, the repetition by the speakers of their own topics indicates a lack of accommodation in discourse management. One can also see a lack of accommodation in interpersonal control and interpretability, although this is not as obvious in the visualisation. Nevertheless, the visualisation makes obvious the lower overall level of accommodation and engagement in this passage. At the level of the whole conversation, the level of engagement was also limited; instead, a large degree of repetition by the patient was observed, indicated by the presence of many red blocks. Furthermore, the doctor’s discourse framed the consultation rather than the patient’s, as it was the doctor’s initial turns that recurred (vertical stripes) throughout the remainder of the consultation.

**Figure 4:**
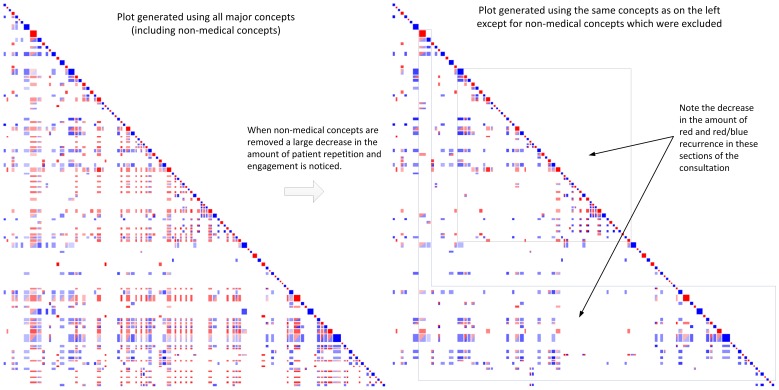
Features of a poor task focussed but good rapport building doctor/patient consultation. Good engagement between the doctor (blue) and patient (red) is observed if non-medical concepts are included (left-hand plot); however removing non-medical concepts highlights how the consultation does not contain good engagement on medical concepts (right-hand plot).

For training consultation #3, where there was poor task focus but good rapport, two separate recurrence plots were generated (see Figure 4). As the conversation was observed to contain a large number of concepts related to sailing (a non-medical topic), one plot was generated using all concepts, and a second plot was generated using only medically relevant concepts. The effect of limiting the available concepts was a reduction in the amount of off-diagonal recurrence, particularly that of the patient. The plot that contained all concepts including those around sailing showed a high degree of engagement, but the recurrences did not stem from medically relevant conversation. The plot that was limited to only medically relevant concepts, including discussion of symptoms (dizziness, nausea), changes in personal situation (work, moving, Hobart) and personal circumstances (wife, family) showed a similar level and distribution of engagement and repetition to Training #2. For example, the vertical stripe stemming from one of the patient’s opening turns was not present after removal of non-medical concepts, indicating that there was no setting of an agenda around the medical issues.

As noted in the literature review, in a doctor/patient consultation the distribution of turn taking needs to match the needs of both interactants and reflects the level of engagement by the patient. Of course, there is not only a single pattern of turn taking. Rather, consultations vary in complexity, type of problem (e.g., chronic versus acute conditions), familiarity by the patient with the problem, and so forth, all of which change the turn-taking pattern. Even so, there are clear types of turn-taking that are more appropriate than others across a range of medical consultations.

In the Discursis visualisations, the size of the on-diagonal squares in the recurrence plots represents the length of the turns (usually the number of words). A general observation about Training consultation #1 was that the patient had many large turns early, and the doctor many large turns later in the consultation. In addition, the distribution of time was shared evenly between both participants (accommodation in discourse management). In the other training examples (Training #2 and Training #3) the doctor contributed the majority of content earlier and later in the consultation. The patient contributed mostly in the middle, or else very little for the entire consultation, indicating a lack of sharing in the management of the discourse.

### Clinical Consultations

#### Clinical #1

**Figure 5:**
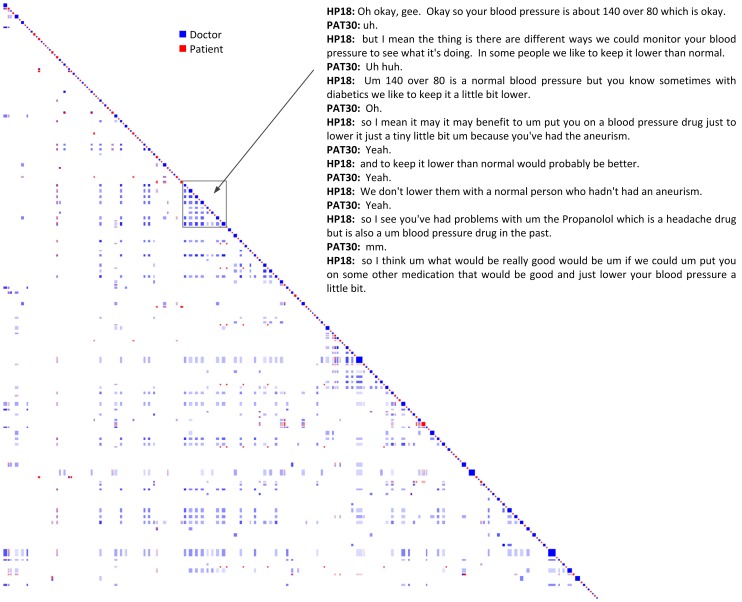
Clinical Dataset #1 (Doctor  =  blue, Patient  =  red). In this dataset the doctor is observed to repeat their own concepts for a large part of the consultation.

As noted above, all patients in the study provided positive ratings of their consultation with the doctor. However, the patient in Clinical #1 gave different post consultation ratings from the majority, in that she was neutral about seeing another doctor next time. She also commented that she felt constrained by time during the consultation, which may have had an impact on her behaviour. For Clinical #1, the recurrence plot indicates a high degree of topic repetition by the doctor on turn-by-turn, medium and long time scales, indicated by the many off-diagonal blocks of blue, as well as a low degree of topic engagement between the patient and doctor (red/blue blocks), or topic repetition by the patient (red blocks). Relative to other consultations, thus, this one exhibited a lower level of same-saying or approximation. One section of interest is highlighted in Figure 5, where there was a large number of blue blocks close to the diagonal but an absence of red and red/blue blocks. This section of the consultation involved the doctor explaining aspects of the patient’s blood pressure, and the patient responding with backchannel replies such as ‘yeah’ and ‘mm’. During this time period, the patient did not mention anything connected to the concept of blood pressure. It was only when the doctor changed the topic to headaches that the patient was able to clarify her understanding of the issues relating to blood pressure. This recurrence pattern was repeated at many locations throughout the consultation, and indicates that the patient did not reuse concepts that were used by the doctor. This observation leads us to conclude that the patient may have left this consultation with little comprehension of what the doctor said. It may be that the doctor did not allow her time to respond appropriately, which would agree with her post consultation rating that she felt constrained by time limitations.

#### Clinical #2

In the post-consultation survey for Clinical #2, the patient provided high ratings of satisfaction with the doctor, but also felt that the doctor had not understood her needs. Given this response, we would expect to see the presence of features similar to those observed in both the good and poor training examples.

**Figure 6:**
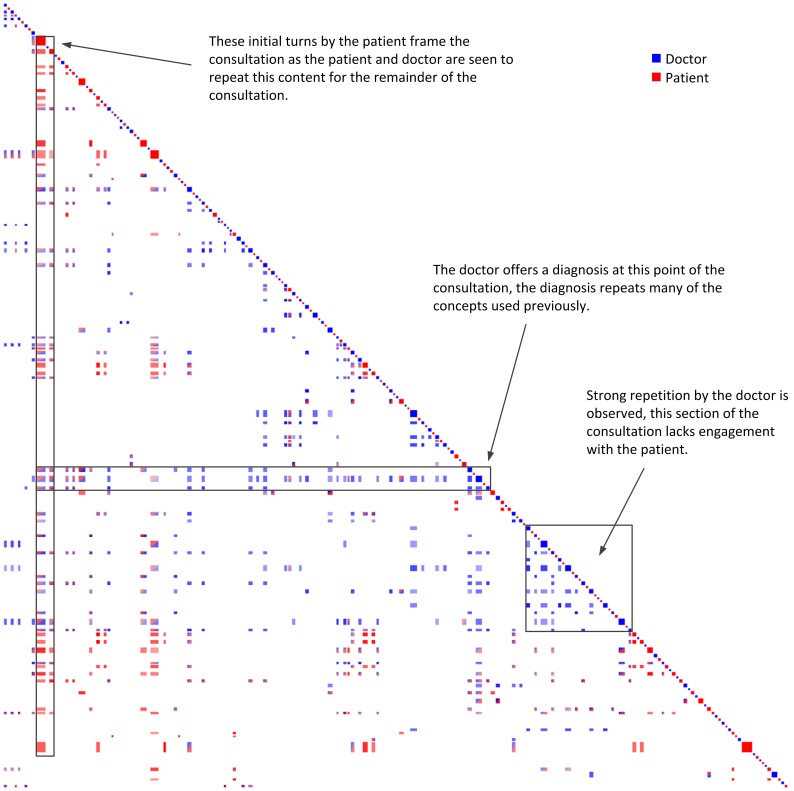
Clinical Dataset #2 (Doctor  =  blue, Patient  =  red). In this dataset the patient’s initial statements recur throughout the entire consultation much like that of Training #1.

The first features of note in Clinical #2 are the vertical stripes stemming from the patient that occur early in the consultation. Similar patterns of recurrence were observed in Training #1, and these stripes indicate that the patient expanded on her issues early in the consultation. Both the patient and the doctor engaged with these issues throughout the consultation. These vertical stripes are indicated in Figure 6, and the text for the first two stripes is reproduced below:

HP22: And um how can I help today.

PAT38: I’m just due for a depo and I just want you to check my ear. Last week it sort of popped but um it stayed that it didn’t pop you know and it took all day for it to sort of um to pop, it was just like I couldn’t hear properly out of it.

HP22: Right.

PAT38: So we’re going to New Zealand in a few weeks so I don’t want to have an ear infection.

Horizontal stripes stemming from the doctor in the later part of the consultation are also similar to those observed in Training #1 when the doctor was offering the patient a diagnosis. These horizontal stripes are indicated in Figure 6, and inspection of the text confirms that these stripes do correspond to a diagnosis by the doctor:

HP22: Now for the ear, what we normally, most of the time between nose and the ear is a tube.

PAT38: ok.

HP22: which controls the pressure, ear, you know fluids going in and out so basically what normally I recommend like using some nasal spray.

PAT38: Oh right Yeah.

HP22: that sometimes helps um the blockage in the ear.

The consultation continues beyond this diagnosis and at this point of the consultation we observe a high degree of repetition by the doctor but limited engagement between the doctor and patient. In terms of CAT, there was accommodation in interpretability, with the doctor making an effort to speak in clear and easy to understand words, but little accommodation in approximation. The lack of engagement in the later part of the consultation may explain why the patient felt that the doctor had not understood her needs. While the doctor offered a medical solution, when this recurrence plot is contrasted with Training #1 and #3, there appears to have been a lack of rapport building.

#### Clinical #3

**Figure 7:**
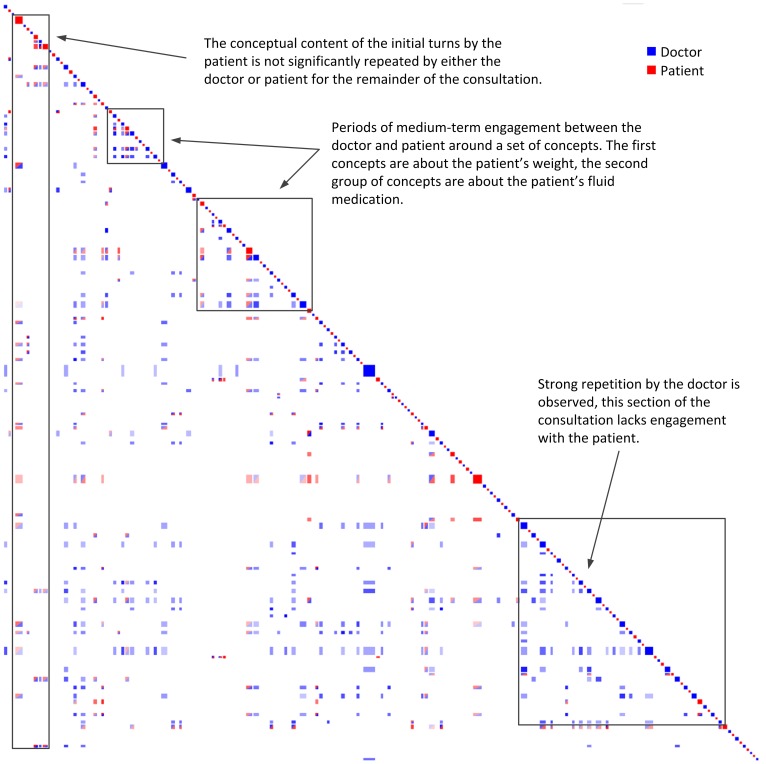
Clinical Dataset #3 (Doctor  =  blue, Patient  =  red). In this consultation we observe small blocks of engagement between the doctor and patient but a lack of a consistent long term agenda.

In Clinical #3 the same doctor (HP22) examined a 39 year old woman who was a pensioner. The patient presented with liver problems, as well as other serious health issues. Again, while this patient provided high satisfaction ratings with the consultation, unlike other patients she did not feel her quality of life had improved a month after the consultation, and she was neutral about feeling better in herself. The recurrence plot for this consultation (see Figure 7) lacks long term structure (evidenced by consistent vertical and horizontal stripes). Instead, it shows more medium and turn-by-turn engagement and repetition. The multitude of health problems that the patient presents may explain the lack of long term structure, as it may have been difficult for the patient to explain all of her problems early in the consultation and address them one by one. The presence of red/blue recurrence close to the diagonal (medium and short-term) suggests that the doctor and patient were engaging well around many of the patient’s health concerns. It also suggests that the doctor was talking to the patient in a language that she could engage with, and the size of the patient’s turns also indicates that she was offering significant detail around these concerns. This consultation is marked by approximation at the conceptual level, even though the conversation involved a large number of different topics.

## Discussion

The aim of this study was to investigate doctor/patient consultations using the Discursis visual text analytic technique. We did this in order to visually identify interaction patterns present in effective consultations, with appropriate communication behaviours by the doctor and the patient. Effective consultations have been shown to be positively correlated with patient satisfaction, treatment adherence and minimization of adverse events [5]. Through the use of short constructed doctor and patient interactions representing exemplars of good and poor rapport and task focus, we were able to visually demonstrate the patterns of interaction that lead to an accurate diagnosis. Vertical stripes of recurrence leading from patient utterances early in the consultation suggest that doctors who encourage patients to expand on the health narrative early are able to obtain details that can be explored for the remainder of the consultation, leading to an accurate diagnosis. Horizontal stripes of recurrence from doctors late in the consultation, which indicate that the major concepts are revisited, have also been linked in previous research to good patient outcomes.

Other findings for the training examples were that doctors who spend too much time on “off-topic”’ banter can build good rapport, but do so at the expense of developing the health narrative; these consultations may demonstrate poor task focus. Allowing a patient to fixate on one set of concepts, rather than addressing all concerns, may result in the narrative halting. This may make it difficult to obtain extra details necessary for an accurate diagnosis. On the other hand, using a check-list style of consultation without reference to the health narrative can hinder the development of the health narrative. In terms of communication accommodation theory, the most effective conversations are characterised by appropriate accommodation in approximation, interpretability, and discourse management in particular.

Having established that Discursis has the facility to detect and display these features, we turned to real life consultations that occurred over a longer time scale and which were of particular interest in the investigation. It is important to remember that clinical studies of doctor/patient communication are confounded by the fact that often doctors who consent to such studies recognise the importance of good communication techniques and tend to be good communicators. For this and other reasons, it was not possible to obtain real life clinical examples that perfectly match the profiles used in the training data. Instead, the three examples selected for analysis were selected from the patients’ feedback on a post-interview questionnaire. While the clinical datasets selected for analysis were all marked as being acceptable by the patients, each patient in our sample indicated that there was one aspect of the consultation with which they were not happy.

The Discursis analysis uncovered specific visual patterns of interaction that may explain why patients felt constrained by time or that doctor had not understood their needs. These patterns included among other things, large sections of single colour recurrence by the doctor. Additionally, we were able to distinguish between doctors who did not balance task and rapport using the Discursis visualisations. In CAT terms, these conversations were all characterised by a lack of accommodation in at least one strategy.

Practitioners can use the Discursis technique to assess their own task and rapport-building competence. Given a transcript, a Discursis plot is easily generated, and visual patterns of interaction can be interpreted in the context of a doctor’s own practice. Many health professionals express a desire to see their own behaviour, and Discursis gives them an efficient means of doing this. Likewise, researchers can use Discursis to analyse large numbers of doctor/patient consultations or other communication datasets, obtaining a close analysis of the text quickly. There is significant potential for exploring and extending communication theories like CAT through a clear visualisation of complex texts.

The analysis in this study was based on the transcribed text alone, ignoring pauses, timing and non-verbal communication. In the future, this kind of meta-data could be added to the Discursis visualisations, and future work could investigate these features and their bearing on communication quality. Discursis might also be used to analyse transcripts coded using techniques such as RIAS (rather than the conceptual coding used in this study) to provide visual interpretations of such coding.

Discursis also has the facility to aggregate the quantity and distribution of the recurrence blocks and assign numeric scores to turns and speakers in a conversation. Using this aggregation facility, Discursis can score speakers based on how often they repeat their own conceptual content in the short, medium or long term, or how much they engage with other speakers’ content. These metrics provide researchers with a useful way to summarise the qualities of a communication in cases where they may have hundreds of transcripts. Possible future work would involve using these metrics to automatically score a large corpus of doctor/patient consultation transcripts and to investigate regularities and variations from them. Overall, Discursis enables a close analysis of medical interactions (and other interactions) with many of the good features of process analysis and microanalysis. This analysis, grounded in the interactions themselves, can be accomplished efficiently across a large corpus of interactions, which can be difficult in manual analyses. In doing this, researchers and practitioners can achieve an important goal in health communication: understanding the impact of task focus and engagement in medical and health outcomes.

## Supporting Information

File S1 Detailed description of the Discursis information visualisation technique, including pre-processing of input text, concept model creation, utterance tagging, graphics generation, and limitations.
(DOC)
