# Supplementary material for: Visualising Conversation Structure across Time: Insights into Effective Doctor-Patient Consultations
Source: PLoS One. 2012 Jun 5;7(6):e38014. doi: 10.1371/journal.pone.0038014 (PMC3367908; doi:10.1371/journal.pone.0038014)
Supplement: File S1 — Detailed description of the Discursis information visualisation technique, including pre-processing of input text, concept model creation, utterance tagging, graphics generation, and limitations. (DOC) [file pone.0038014.s001.doc]

## Data Analysis: Discursis Information Visualisation Technique

Discursis processes an input transcript in five major steps: text input; text pre-processing; concept building; conceptual tagging of conversation turns; and, graphics generation (described briefly in the following sections). For complete details of algorithms used in this process .

### Text Input

The training and clinical transcripts used in this study focus only on transcribed spoken dialogue and do not include gesture or other meta-information. The text transcripts used in this study, both training and clinical, were formatted as comma separated value (CSV) files. Each individual line in the CSV file indicates a turn taken by a conversation participant, and commas were used to separate columns of information. Each file contains two columns: the first is the participant ID, and the second is the spoken text. Below is an example from a clinical consultation between HP02 (doctor) and PAT02 (patient), containing four turns by two participants:

HP02, “Right and you're married.”

PAT02, “Yes.”

HP02, “Children.”

PAT02, “No children as yet.”

### Text Pre-processing

Conversation transcripts are processed one at a time by the visualisation system. For visualisation purposes a turn is defined as a line of text input; as such, a turn may contain a few words, multiple sentences or even paragraphs of spoken text.

The data structure for each turn contains a copy of the original spoken text, in addition to a processed version of the text that does not include stop words. Stop words include articles, prepositions, common words and vocal gestures (hmm, uh, huh, etc.)[[1]](#footnote-2). As the text input has an implicit ordering (but no other timing information) each turn was numbered in the order that it was spoken.

### Concept Building

The processed text from all turns (within a single conversation file) was combined to create a semantic model that was later used to tag turns based on their individual conceptual content. The semantic model uses word occurrence and co-occurrence information to infer connections between individual terms. This semantic model makes it possible to measure any two terms’ semantic similarity (defined here as extent of co-occurrence, or extent of occurrence in the same context). Concretely this means that if two terms are used interchangeably throughout a text, (they share similar contexts), then those terms will have high semantic similarity.

Many algorithms could be used to determine semantic similarity. Some algorithms are based on relative frequencies of terms, as first introduced by Salton (1989). Others employ a range of more complex methods, including Leximancer , Latent Semantic Analysis and Latent Dirichlet Allocation . For this study, semantic similarity was calculated using the statistical technique introduced by . The technique was selected for its high fidelity with small document input and for its simplicity of use. We refer readers to the original reference , or to for complete details of the algorithm.

Salton’s semantic algorithm provides a subset of key terms (concepts), which are the most common terms in the input text. For this study we used the top 50 concepts from each input text, instead of using a pre-defined ontology or thesaurus.

### Concept Tagging

Each turn in the input data was tagged according to how the processed text matched the top 50 concepts. Concretely this means that each turn contained a vector of numeric values with each value corresponding to a concept from the 50 top concepts. If the processed text of a turn contained terms that had a high semantic similarity to particular concepts, then those concepts had a high concept similarity score.

The concept vectors for each turn form the basis of the visualisation system. Given that each turn is translated into a vector of values with a common basis (50 top concepts), they can be compared directly to see if they contain a similar mixture of concepts.

### Graphics Generation

The conceptual recurrence plot in Discursis is a visualisation technique based on a matrix of size *n × n*, where *n* is the number of turns in the conversation under study. The diagonal elements of the matrix represent each turn, and the off-diagonal elements represent the conceptual similarity of the horizontally and vertically adjacent turns. Only one side of the diagonal is shaded due to the symmetric nature of the comparisons being made.

The conceptual similarity of any two turns (the off-diagonal shading) is measured by comparing the concept vectors of these two turns using a vector dot product. The dot product returns a value between 0 and 1, a value of 0 meaning that the turns share no conceptual content, and a value of 1 meaning that these turns are conceptually identical. The off-diagonal elements (recurrence elements) are rendered by shading each element according to this score (1 = boldest colour, 0 = white).

Block sizing in the recurrence plot was set proportional to the number of words spoken in the turn. Different colours are used to indicate specific speakers within the conversation. In all datasets analysed in this study, only two participants are present. Thus one is tagged as red and the other is tagged as blue. Each off-diagonal element of the recurrence plot is coloured in the speaker-specific colour if it corresponds to a conceptual comparison between two turns by this same speaker. If an off-diagonal element corresponds to a conceptual comparison between the two different speakers, a gradient of colour is used. An example conceptual recurrence plot generated from a subset of text from a Doctor/Patient consultation is shown in Figure 1.

### Abilities and Limitations

The conceptual matching algorithm used by Discursis relies on the collocation of terms within an input text for determining conceptual similarity. The text transcript itself is used to generate a semantic model. If two terms are frequently mentioned in temporal proximity in this text transcript, they will be strongly connected; if they are never collocated, they will have no semantic connection. Concepts are invoked strongly when the concept itself is mentioned’ this is the case with the words “times,” “day,” and “smoked” (conceptual matches in Figure 1). In the same example, the third last and last turns (both by the patient) are also similar, even though they share no terms in common.[[2]](#footnote-3) Instead, in this example the terms “week” and “nights” relate to similar concepts. The concept approach of Discursis is useful for locating when similar concepts are evoked even though different words are used, but Discursis does not currently handle functional connections, one example being a positive or negative affirmation to a question. For example, if a physician asked a question to a patient and received a response “no”, “yes” or “I guess”, it is unlikely that these turns would be matched, whereas if the patient offered a detailed response including terms that related to similar concepts used by the physician, the turns would be connected (e.g., the last two turns in Figure 1).

## References

1. Angus D, Smith AE, Wiles J (2012) Conceptual recurrence plots: Revealing patterns in human discourse. IEEE Transactions on Visualization and Computer Graphics 18: 988 - 997.

2. Angus D, Smith A, Wiles J (2012) Human Communication as Coupled Time Series: Quantifying Multi-participant Recurrence. IEEE Transactions on Audio, Speech, and Language Processing 20: 1795 - 1807.

3. Smith AE, Humphreys MS (2006) Evaluation of unsupervised semantic mapping of natural language with Leximancer concept mapping. Behavior Research Methods 38: 262-279.

4. Landauer TK, Foltz PW, Laham D (1998) An introduction to latent semantic analysis. Discourse processes 25: 259-284.

5. Blei DM, Ng AY, Jordan MI (2003) Latent dirichlet allocation. The Journal of Machine Learning Research 3: 993-1022.

6. Salton G (1989) Automatic text processing: the transformation. Analysis and Retrieval of Information by Computer.

1. The stop word list used in this study can be found at: [http://www.itee.uq.edu.au/ uqdangus/stopwords.txt](http://www.itee.uq.edu.au/~uqdangus/stopwords.txt) [↑](#footnote-ref-2)
2. “I”, “have” and “a” are all stop words and are not used in generating conceptual matches. [↑](#footnote-ref-3)
